# Aminode: Identification of Evolutionary Constraints in the Human Proteome

**DOI:** 10.1038/s41598-018-19744-w

**Published:** 2018-01-22

**Authors:** Kevin T. Chang, Junyan Guo, Alberto di Ronza, Marco Sardiello

**Affiliations:** 1 Department of Molecular and Human Genetics, Baylor College of Medicine, Jan and Dan Duncan Neurological Research Institute, Texas Children’s Hospital, Houston, TX 77030 USA; 2 Present Address: Microsoft Corporation, 1 Microsoft Way, Redmond, WA 98052 USA

## Abstract

Evolutionarily constrained regions (ECRs) are a hallmark for sites of critical importance for a protein’s structure or function. ECRs can be inferred by comparing the amino acid sequences from multiple protein homologs in the context of the evolutionary relationships that link the analyzed proteins. The compilation and analysis of the datasets required to infer ECRs, however, are time consuming and require skills in coding and bioinformatics, which can limit the use of ECR analysis in the biomedical community. Here, we developed Aminode, a user-friendly webtool for the routine and rapid inference of ECRs. Aminode is pre-loaded with the results of the analysis of the whole human proteome compared with proteomes from 62 additional vertebrate species. Profiles of the relative rates of amino acid substitution and ECR maps of human proteins are available for immediate search and download on the Aminode website. Aminode can also be used for custom analyses of protein families of interest. Interestingly, mapping of known missense variants shows great enrichment of pathogenic variants and depletion of non-pathogenic variants in Aminode-generated ECRs, suggesting that ECR analysis may help evaluate the potential pathogenicity of variants of unknown significance. Aminode is freely available at http://www.aminode.org.

## Introduction

Evolutionary changes along a protein sequence occur at rates that are inversely correlated with the strength of specific constraints at each site. Constrained regions are considered to be under functional constraint owing to a role in protein stability, post-translational modifications, subcellular localization, interaction with other molecules, or enzymatic function^1–4^. Because constraint can vary widely along a given protein sequence, profiling the rates of evolutionary changes can provide information useful to identify the key residues or domains of the protein.

Several studies have shown that evolutionarily constrained regions (ECRs) can pinpoint the position of residues that are relevant for the function of enzymes or other protein types and can even provide significant information to predict the effects of specific mutations^5–11^. Therefore, the identification of ECRs may help inform investigation and experimental design of protein studies. For example, profiling evolutionary constraint can indicate regions to avoid or to target for protein tagging when the function or interactions of the protein must be preserved. Conversely, highly constrained regions might be an excellent choice for functional studies based on mutagenesis analysis^7,8,12^. In the absence of prior experimental data, the identification of ECRs may indeed point towards candidate positions in a protein that, if mutated, may have a deleterious effect on the protein function. The underlying reasoning is that if a site has been refractory to changes over long periods of evolutionary time—as inferred from a comparison of numerous and distantly related taxa—any change at that site is likely deleterious^13,14^.

Effective methods of profiling a set of homologous proteins to determine ECRs require the simultaneous analysis of amino acid sequences and phylogenetic relationships of the proteins under examination^15,16^. A general approach to identify ECRs consists of a multi-step procedure^15^: First, orthologs of the protein of interest are selected and a multiple alignment is generated to allow the measurement of the relative rate of substitution at each protein position. Depending on the analysis to be performed, paralogs may also be included—closely related paralogs if the analysis is focused on specific structural features of the protein under examination, or both close and distant paralogs if the analysis is aimed at identifying general constraints of the protein family^5,15^. Next, the number of substitutions that have occurred at each protein position is computed based on the phylogenetic relationships among the proteins under examination; the information is then used to calculate the relative rate of substitutions in a sliding window of a fixed length over the entire protein multiple alignment, where each window’s relative rate is obtained by dividing the substitution rate in that window by the average of all windows. Relative rates are finally plotted as a function of their position along the protein alignment, and ECRs are identified as corresponding to the “valleys” in the plot^15^. These procedures are time consuming and require skills in coding and bioinformatics. As a service to the biomedical community, we have developed a web tool, Aminode, which automatically profiles protein evolutionary constraints with a minimal amount of information from the user. Aminode is freely available, includes a pre-computed analysis of the human proteome, and allows download of high-resolution graphs and computed data for immediate use.

## Results

### Aminode Scope

Aminode calculates the relative amino acid substitution rates of the protein(s) of interest and identifies evolutionarily constrained regions (ECRs) via a comparative analysis of multiple protein homologs in the context of their evolutionary relationships. The Aminode pipeline performs analyses based on two inputs: (i) The amino acid sequences of the protein homologs, and (ii) a phylogenetic tree that describes the evolutionary relationships of the inputted protein homologs. Aminode implements a user-friendly, web-based interface that allows two modalities of analysis:

#### Pre-computed analysis of the human proteome

Users can retrieve the results from the pre-computed analysis of the human proteome cross-analyzed against 62 vertebrate proteomes available in Ensembl genome browser^17^. For this analysis, the Aminode pipeline was executed using annotated vertebrate orthologs of human proteins, which resulted in the determination of the relative amino acid substitution rates and the identification of evolutionary constrained regions for a total of 18,713 human proteins.

#### Custom analysis

Users can analyze their proteins of interest by submitting the proteins’ amino acid sequences and, optionally, their phylogenetic tree to profile the rate of evolutionary changes and identify ECRs using customizable parameters.

### The Aminode Pipeline

**Figure 1:**
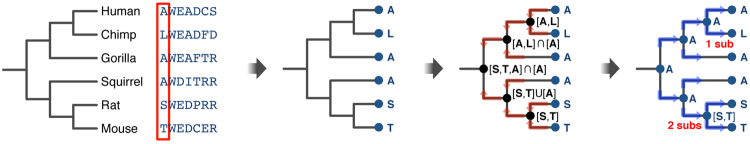
An example of the procedure used to compute ancestor nodes based on the Hartigan algorithm.

**Figure 2:**
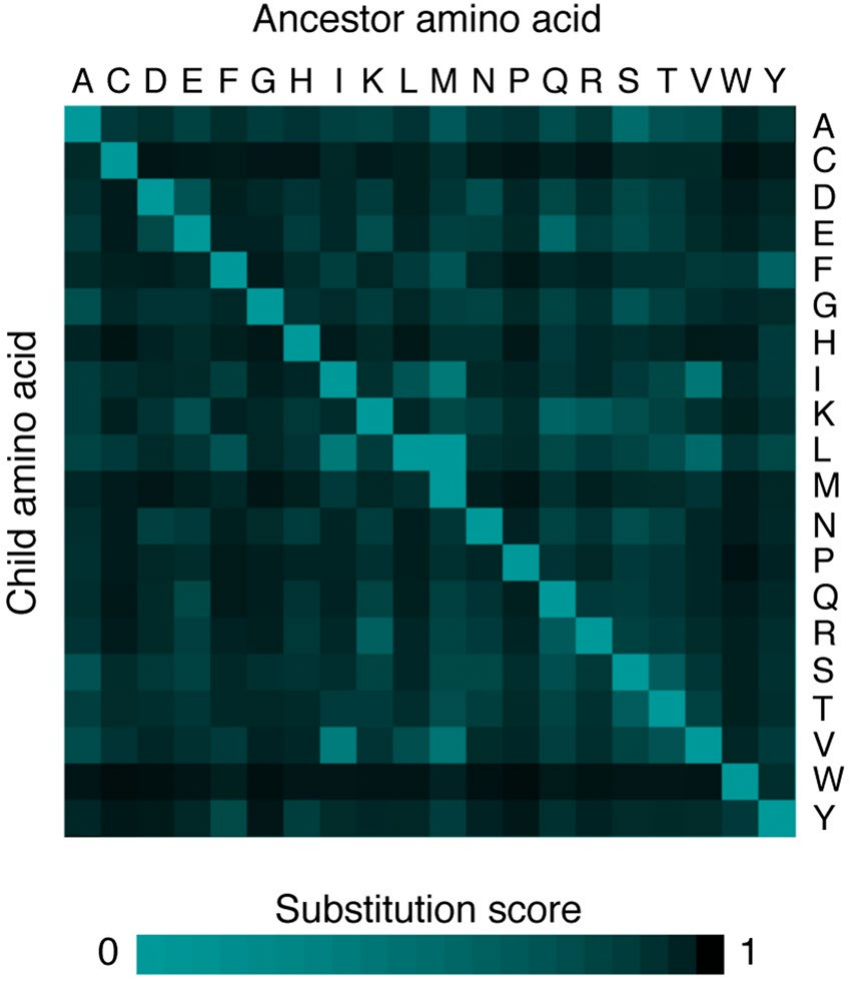
Heatmap of the ancestor-to-child amino acid substitution scores used in the Aminode pipeline.

Protein multiple alignments are obtained by using Multalin^18^ (http://multalin.toulouse.inra.fr/multalin/) with default parameters. Columns containing gaps in more than 50% of aligned proteins are eliminated from the multiple sequence alignment. The phylogenetic tree is converted to a noded tree where the end nodes are the current species used in the analysis, and the ancestor nodes represent the last common ancestor of each branch. The Hartigan algorithm provides a framework for calculating best fits of a given tree according to a maximum parsimony approach^19^ and is here used for calculating the minimum mutation fits at all aligned amino acid positions. Briefly, according to the parsimony criterion, the algorithm seeks a phylogenetic history that explains tree topology and/or amino acid changes with the fewest number of evolutionary events. In the Aminode pipeline, the tree topology is either fixed (the pre-computed analysis of the human proteome is based on comparison with species with known phylogenetic relationships) or calculated based on the input sequences in custom analyses (see below). Thus, in Aminode the Hartigan algorithm was used to infer amino acid identities in the ancestral nodes of the given evolutionary tree. In particular, for each amino acid position, a bottom-up procedure compares the amino acids from the child nodes to their immediate ancestral node and establishes that each ancestral node is equal to the intersection of its child nodes if the intersection is not empty (that is, if the child nodes share the same amino acid); otherwise, it is equal to their union (see example in Fig. 1). The subsequent top-down refinement retains, at each node, the amino acid that gives the minimum node substitution score^19^ (NSS) (Fig. 1), which is assigned based on a modified BLOSUM62 Target Frequencies matrix^20^ available at the NIH Repository (ftp://ftp.ncbi.nih.gov/repository/blocks/unix/blosum). We obtained a scoring system in which node substitution scores can vary from 0 to 1, with 0 denoting no changes (amino acid self-substitution), scores increasing with the rarity of BLOSUM62 substitution occurrence, and 1 as a theoretical maximum (amino acid substitution observed zero times). This was obtained by normalizing to 1 the sum of the frequencies of substitution (F_S_) for each amino acid (including self-substitution, F_SS_) to take into account differences in amino acid abundances, and then by calculating each node substitution score as NSS = 1 − (F_S_/F_SS_). A graphical representation of the matrix of amino acid substitution scores is reported in Fig. 2. For each position of the multiple alignment, a substitution score (SS) is calculated as the sum of all node substitution scores at that position. A relative substitution score is then obtained by diving the SS by the number of informative sequences (no. of sequences with an amino acid in that position); these values are finally normalized by the mean relative substitution score, and then averaged by using an 11-amino acid-long sliding window across the whole protein length, with two consecutive smoothing steps using a 7-amino acid-long sliding window^5^. The resulting profile describing the weighted relative rate of amino acid substitution is plotted as a function of alignment position in a two-dimensional array using the JFreeChart Java library (http://www.jfree.org/jfreechart). Scanning the array from the bottom (minimum) to the top (maximum) leads to the identification of local minima or evolutionarily constrained regions (ECRs), whose extent is defined by the closest proximal and distal positions where the second derivative of the plot is zero. Computed data are transferred to Excel files using the Apache POI Java library (https://poi.apache.org/) and are available for download.

To execute the analysis of the human proteome, the protein sequences and phylogenetic tree of 63 species (human plus 62 additional vertebrate species) were downloaded from the Ensembl genome browser^17^ (release 84), and the Aminode pipeline was executed on each ortholog series. Proteins containing long stretches of incomplete sequences or long out-of-frame regions possibly derived from annotation errors were excluded from the analysis.

### Backend Information

The Aminode website (www.aminode.org) is hosted on Heroku and uses the Spark Framework for the web server. Precomputed files are hosted on Microsoft Azure Storage and Github using Large File Storage (LFS), and bulk data are hosted on Google Drive. All files are retrieved via Aminode Search through link generation. The frontend uses the AngularJS framework, and the backend uses Java to process data and generate various output files.

### Aminode Content

**Figure 3:**
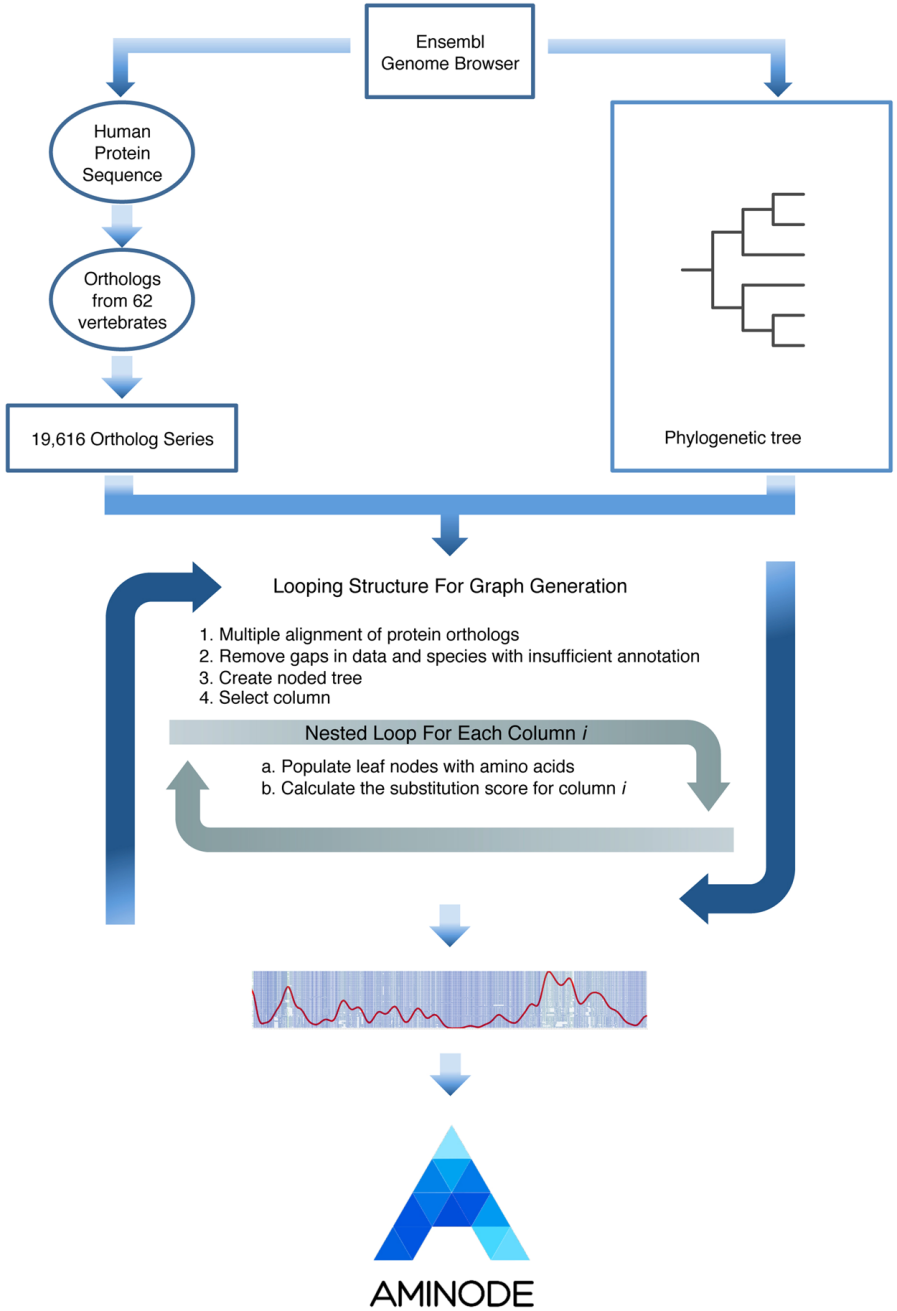
Schematic of the pipeline for the generation of Aminode graphs.

Aminode contains results from evolutionary constrained region analyses for human proteins that have at least two vertebrate orthologs annotated in Ensembl, Release 84 (18,713 proteins). Figure 3 shows the pipeline for the generation of Aminode graphs. Aminode pre-generated outputs provide a visual representation of the relative rate of amino acid substitution as a line plotted over the multiple sequence alignment (one example is reported below). Local minima indicate regions with low rates of substitution relative to the surrounding protein regions, while maxima indicate relative high rates. The valleys in the graphical output therefore indicate protein regions that are evolutionarily more constrained than the regions identified by the peaks. The positions of the predicted ECRs are marked by yellow bars placed above the multiple alignment. As a reference, the human protein index is reported on the top of the multiple alignment.

Aminode is searchable by the HGNC designated gene name (standard gene symbol). Full-resolution images and tabulated data can be downloaded from the links provided as result of the search. Computed data is downloaded as an Excel file that contains the information processed to execute the Aminode ECR analysis starting from the protein sequences. This file is provided for maximum ease in further processing of Aminode data. The Excel file includes the following tabs: (1) The “Substitution Scores” tab, which contains the human protein index, the filtered alignment index, the human amino acid sequence, the substitution scores, and the information relative to the relative substitution scores used to generate the Aminode graph (plot of relative substitution scores and ECR indexes). (2) The “Raw Substitution Scores” tab contains the raw aligned index, the human protein index, the human amino acid sequence, and the substitution scores. (3) The “Aligned Sequences” tab contains the full multiple alignment of the ortholog series. At the top of the alignment there are three indexes that track the sequences according to the Human Protein Index, Filtered Aligned Index, and Raw Aligned Index, respectively. The Filtered Aligned Index keeps track of the protein after filtering the data for gaps in the sequences. The Raw Aligned Index keeps track of the protein after the multiple alignment. The Human Protein Index keeps track of the original index of the human protein sequence.

Links to a Gene Summary page (containing information automatically extracted from MyGene.info^21^) and to the queried gene’s entry in other gene information sites, including NCBI^22^, UniProt^23^, and GeneCards^24^ are provided. UniProt ID and NCBI ID are obtained from MyGene.info.

Additional links provide access to the amino acid sequences of all the orthologs used in the queried gene’s Aminode-generated analysis, and to a Github Repository that contains the information generated and used in the Aminode analysis, available for download. In summary, each entry in Aminode provides access to a graph with the protein evolutionary profile plotted over the multiple protein alignment, raw data (original FASTA files), processed files (multiple alignments), list of rates of substitutions, scraped data, and excel files with the processed data formatted and graphed. Text files with bulk data (aligned and non-aligned sequences and relative substitution scores) are also available for download.

### Custom Protein Analysis

The Aminode pipeline is also available to perform analyses with either a different species focus or a custom set of protein sequences. The user is prompted to (i) submit a set of protein sequences in standard FASTA format, and (ii) either submit a phylogenetic tree describing the protein evolutionary relationships in Newick format^25^ or, alternatively, generate the tree via the option offered by Aminode, which uses the Multalin algorithm^18^. The names used to label proteins (or species) in the submitted protein sequence file must match the names of the leaf nodes in the submitted phylogenetic tree. The sequences do not need to be in any specific order. The user can adjust parameters such as filter threshold, font size and graph colors for the generation of the graphical output.

### Mapping amino acid frequencies, post-translational modifications and human missense variants

**Figure 4:**
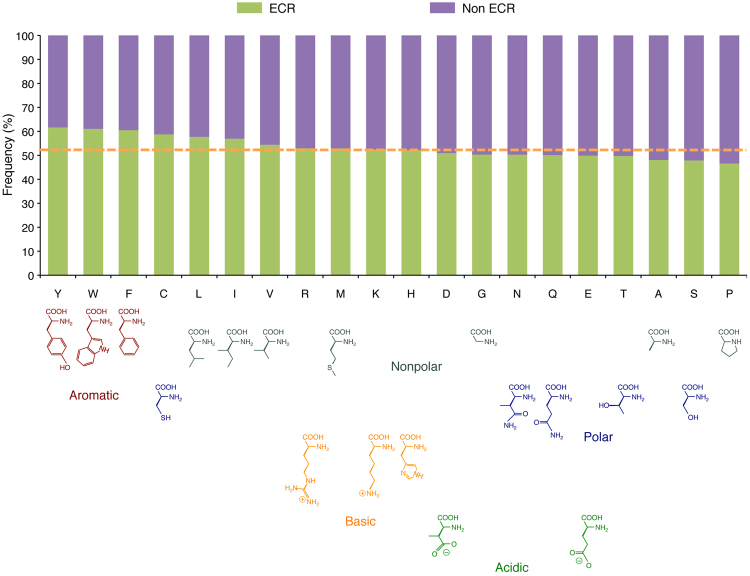
Frequencies of amino acids in evolutionarily constrained regions (ECRs) and non-ECRs across the human proteome. Amino acids are arranged left to right accordingly to their decreasing ECR/non-ECR frequency. The orange dotted line indicates the frequency expected by random distribution.

**Figure 5:**
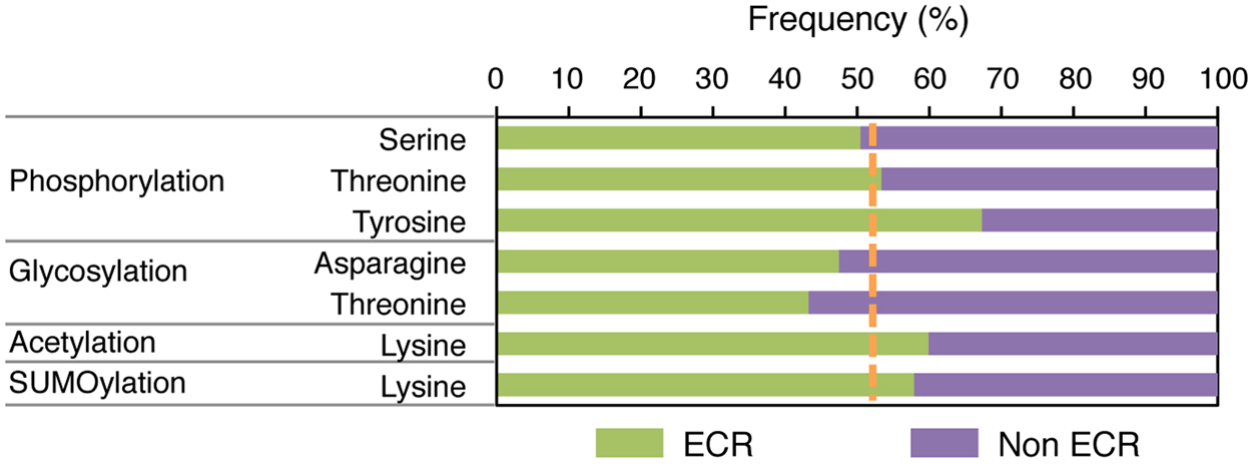
Frequencies of phosphorylated, glycosylated, acetylated and SUMOylated amino acids in evolutionarily constrained regions (ECRs) and non-ECRs across the human proteome. The orange dotted line indicates the frequency expected by random distribution.

We investigated whether ECRs have different amino acid composition from non-ECRs by examining amino acid frequencies in ECRs and non-ECRs. The analysis showed that the aromatic amino acids (tyrosine, tryptophan and phenylalanine) have the most skewed distribution, showing a significant enrichment in ECRs (Bonferroni-adjusted Fisher’s *P* < 10^−4^ for all) (Fig. 4). Conversely, in the group of polar amino acids, serine, threonine, glutamine and asparagine are depleted, while cysteine is enriched, in ECRs (*P* < 10^−4^ for all). The distribution of basic amino acid does not differ between ECRs and non-ECRs, while both glutamic acid and aspartic acid are depleted in ECRs (*P* < 10^−4^ for both). Finally, among non-polar amino acids, leucine, isoleucine and valine are enriched, while alanine and proline are depleted, in ECRs (*P* < 10^−4^ for all) (Fig. 4). We also examined the distribution of annotated sites^23^ of the most common types of post-translational modification. While phosphoserine and phosphothreonine do not show skewed distribution, phosphotyrosine is enriched in ECRs (*P* < 10^−4^) (Fig. 5), and all three have slightly increased frequencies in ECRs compared to their non-phosphorylated counterparts (*P* < 10^−4^ for all), which may indicate structural or functional relevance for some of these sites. Acetylated or SUMOylated lysine also shows enrichment in ECRs (*P* < 10^−4^ for both). Conversely, both glycosylated asparagine and threonine show significant depletion from ECRs, indicating relatively smaller structural conservation despite glycosylation relies on a target motif that extends shortly beyond the target amino acid^26^. This effect may depend on the fact that glycans, rather than amino acids, may direct protein interaction or function at the modified sites, or on a masking effect that bulky glycans may exert on the nearby amino acids, thus making them less available to selection-driving interactions.

**Figure 6:**
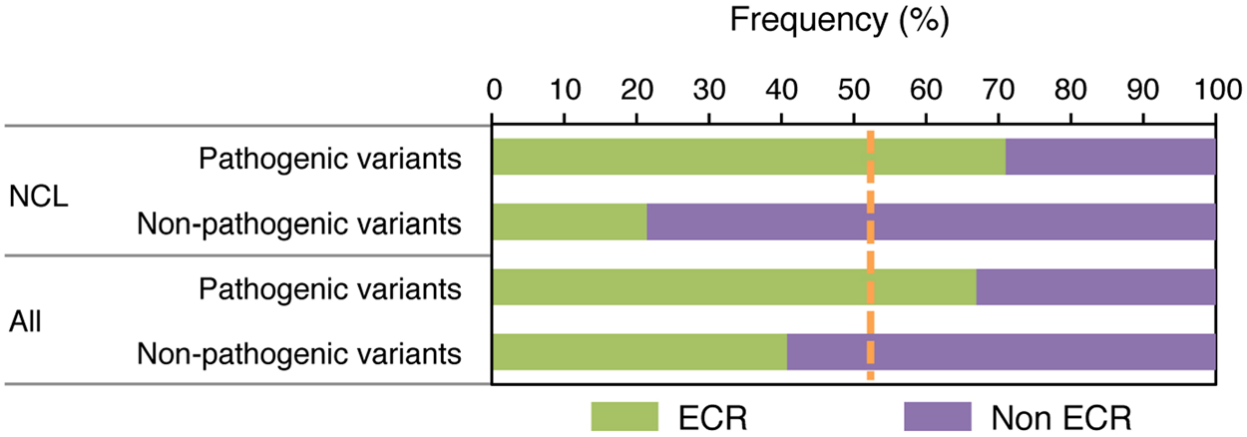
Distribution of pathogenic and non-pathogenic variants described in neuronal ceroid lipofuscinosis (NCL) proteins and in the human annotated proteome in evolutionarily constrained regions (ECRs) and non-ECRs. The orange dotted line indicates the frequency expected by random distribution.

We also investigated the distribution of known human missense variants in ECRs by examining the lists of pathogenic and nonpathogenic variants reported in UniProt^23^. We first focused on a group of neurodegenerative diseases named neuronal ceroid lipofuscinoses or Batten disease, for which high-quality annotations of pathogenic mutations are available^27^. Interestingly, 71% of annotated pathogenic missense mutations in Batten disease proteins map in ECRs, compared to 21% of nonpathogenic variants (*P* < 10^−4^) (Fig. 6). Similarly, 67% of reported pathogenic variants across the entire proteome were found to fall within ECRs, compared to 41% of non-pathogenic variants (*P* < 10^−4^).

## Discussion

ECR analysis may help pinpoint protein sites that are under purifying selection over a certain evolutionary time scale. The selection maintains conservation at sites crucial to structure and function of the protein. Thus, from such observed evolutionary constraints one may deduce and predict the relative importance of specific protein sites^1–4^. Aminode enables the execution of complex sequence analyses in order to identify protein regions that are either evolutionarily constrained or unconstrained. The Aminode webtool allows researchers the swift identification of ECRs in proteins of interest and specifically provides the results of evolutionarily constrained region analyses for vertebrate proteome data available from Ensembl with a focus on the human proteome. In addition, Aminode enables user-customized analyses for proteins of interest.

**Figure 7:**
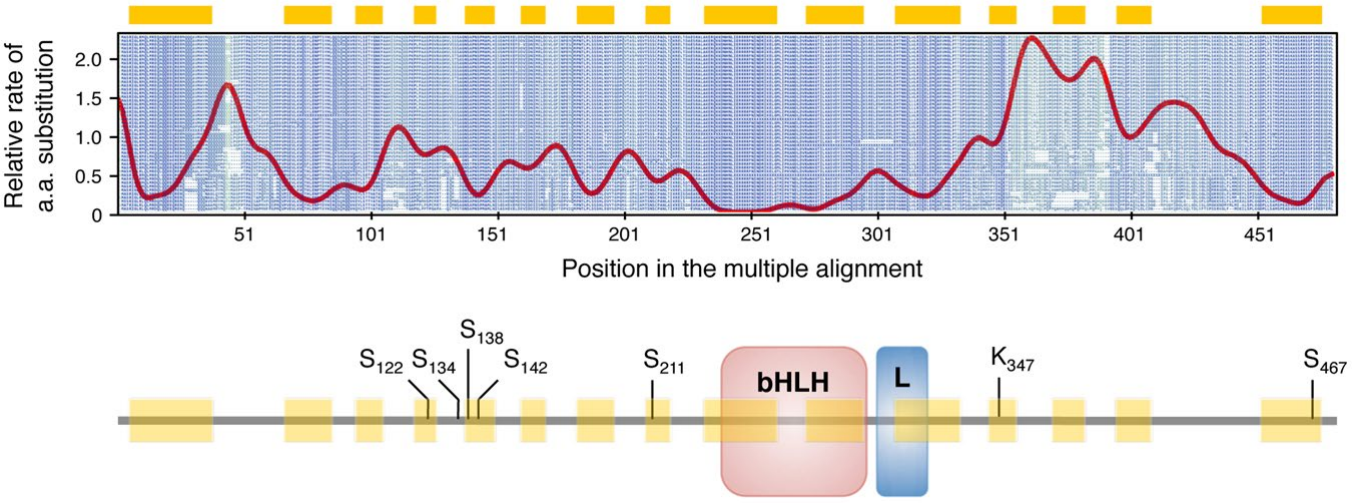
Aminode example: Analysis of transcription factor EB (TFEB). *Upper panel*, Aminode graphical output. The red line represents the relative rate of amino acid substitution calculated at each protein position. Local minima (highlighted by yellow bars above the graph) are constrained regions with relatively low substitution rates. Peaks (local maxima) indicate regions with relatively high substitution rates. The amino acid sequences for each ortholog are shown in shades of blue to green (more conserved to less conserved) in the background of the graph. *Lower panel*, Schematic of TFEB protein structure showing the position of the DNA-binding bHLH domain, the leucine zipper domain (L) and various known sites of post-translational modification that regulate TFEB function (references in the text).

The potential importance of *in silico* support for ECRs is multifold. First, ECRs can predict functional importance, providing researchers with key information to design their bench experiments. ECRs may indeed contain residues that are part of the active site in enzymes, map sites that are essential to the protein structure or function, and help identify post-translational modification sites^5–11^. The example reported in Fig. 7 represents the analysis of the transcription factor EB (TFEB), a master transcriptional regulator of lysosomal degradative pathways^28–30^ that is being studied in our laboratory. The example reports a schematic of the structure of TFEB and shows that the DNA-binding bHLH domain, the leucine zipper domain, and six out of seven experimentally validated post-translational modification sites of TFEB that regulate TFEB function^31–38^ fall within Aminode-identified ECRs (Fig. 7), underlying the overlap between ECRs and functionally relevant sites.

Second, researchers executing experiments of protein manipulation could benefit from Aminode use. Terminal or internal protein tagging can be designed on the basis of Aminode analyses to select unconstrained regions to minimize the potential impact of the tag to the protein’s function or interactions; conversely, targeted disruption of constrained regions may be used to experimentally identify essential protein sites. The identification of ECRs could also be useful to evaluate the potential impact of vector insertions in large-scale mutagenesis projects^39–44^.

Third, due to the observed tendency of pathogenic variants to fall within ECRs, Aminode can serve as a tool to help evaluate which variants of unknown significance are more likely to be pathogenic and/or require further investigation. The integration of Aminode analysis with that of tools such as PhastCons^45^ and PhyloP^46^, which investigate evolutionary conservation at the nucleotide level, may provide a wider perspective on the potential impact of variants that cause changes in the amino acid sequence of a protein.

Aminode will be continuously updated as genome assemblies are updated and newly sequenced genomes become available and curated in Ensembl.

### Data Availability

All data generated or analyzed during this study are available at the Aminode website: http://www.aminode.org.

## References

[1] Lichtarge O, Bourne HR, Cohen FE (1996). An evolutionary trace method defines binding surfaces common to protein families. J Mol Biol.

[2] Lichtarge O, Bourne HR, Cohen FE (1996). Evolutionarily conserved Galphabetagamma binding surfaces support a model of the G protein-receptor complex. Proc Natl Acad Sci USA.

[3] Dean, A. M. & Golding, G. B. Enzyme evolution explained (sort of). *Pac Symp Biocomput*, 6–17 (2000). 10.1142/9789814447331_0002 10902152

[4] Karchin R, Cline M, Karplus K (2004). Evaluation of local structure alphabets based on residue burial. Proteins.

[5] Sardiello M, Annunziata I, Roma G, Ballabio A (2005). Sulfatases and sulfatase modifying factors: an exclusive and promiscuous relationship. Hum Mol Genet.

[6] Lunzer M, Golding GB, Dean AM (2010). Pervasive cryptic epistasis in molecular evolution. PLoS Genet.

[7] Ko DC, Binkley J, Sidow A, Scott MP (2003). The integrity of a cholesterol-binding pocket in Niemann-Pick C2 protein is necessary to control lysosome cholesterol levels. Proc Natl Acad Sci USA.

[8] Ota M, Kinoshita K, Nishikawa K (2003). Prediction of catalytic residues in enzymes based on known tertiary structure, stability profile, and sequence conservation. J Mol Biol.

[9] Kashuk CS (2005). Phenotype-genotype correlation in Hirschsprung disease is illuminated by comparative analysis of the RET protein sequence. Proc Natl Acad Sci USA.

[10] Jackson PJ (2006). Structural and molecular evolutionary analysis of Agouti and Agouti-related proteins. Chem Biol.

[11] Lin RJ, Blumenkranz MS, Binkley J, Wu K, Vollrath D (2006). A novel His158Arg mutation in TIMP3 causes a late-onset form of Sorsby fundus dystrophy. Am J Ophthalmol.

[12] Spatuzza C (2008). Physical and functional characterization of the genetic locus of IBtk, an inhibitor of Bruton’s tyrosine kinase: evidence for three protein isoforms of IBtk. Nucleic Acids Res.

[13] Stone EA, Sidow A (2005). Physicochemical constraint violation by missense substitutions mediates impairment of protein function and disease severity. Genome Res.

[14] Binkley J (2010). ProPhylER: a curated online resource for protein function and structure based on evolutionary constraint analyses. Genome Res.

[15] Simon AL, Stone EA, Sidow A (2002). Inference of functional regions in proteins by quantification of evolutionary constraints. Proc Natl Acad Sci USA.

[16] Yang Z, Nielsen R, Goldman N, Pedersen AM (2000). Codon-substitution models for heterogeneous selection pressure at amino acid sites. Genetics.

[17] Yates A (2016). Ensembl 2016. Nucleic Acids Res.

[18] Corpet F (1988). Multiple sequence alignment with hierarchical clustering. Nucleic Acids Res.

[19] Hartigan JA (1973). Minimum Mutation Fits to a Given Tree. Biometrics.

[20] Henikoff S, Henikoff JG (1992). Amino acid substitution matrices from protein blocks. Proc Natl Acad Sci USA.

[21] Xin J (2016). High-performance web services for querying gene and variant annotation. Genome Biol.

[22] Coordinators, N. R. Database Resources of the National Center for Biotechnology Information. *Nucleic Acids Res*, doi:gkw1071 (2016). 10.1093/nar/gkv1290 PMC4702911 26615191

[23] UniProt C (2015). UniProt: a hub for protein information. Nucleic Acids Res.

[24] Stelzer G (2016). The GeneCards Suite: From Gene Data Mining to Disease Genome Sequence Analyses. Curr Protoc Bioinformatics.

[25] Cardona G, Rossello F, Valiente G (2008). Extended Newick: it is time for a standard representation of phylogenetic networks. BMC Bioinformatics.

[26] Ohtsubo K, Marth JD (2006). Glycosylation in cellular mechanisms of health and disease. Cell.

[27] Mole SE, Cotman SL (2015). Genetics of the neuronal ceroid lipofuscinoses (Batten disease). Biochim Biophys Acta.

[28] Sardiello M (2009). A gene network regulating lysosomal biogenesis and function. Science.

[29] Sardiello M (2016). Transcription factor EB: from master coordinator of lysosomal pathways to candidate therapeutic target in degenerative storage diseases. Ann N Y Acad Sci.

[30] Sardiello M, Ballabio A (2009). Lysosomal enhancement: a CLEAR answer to cellular degradative needs. Cell Cycle.

[31] Palmieri M (2017). mTORC1-independent TFEB activation via Akt inhibition promotes cellular clearance in neurodegenerative storage diseases. Nat Commun.

[32] Vega-Rubin-de-Celis S, Pena-Llopis S, Konda M, Brugarolas J (2017). Multistep regulation of TFEB by MTORC1. Autophagy.

[33] Martina JA, Chen Y, Gucek M, Puertollano R (2012). MTORC1 functions as a transcriptional regulator of autophagy by preventing nuclear transport of TFEB. Autophagy.

[34] Roczniak-Ferguson A (2012). The transcription factor TFEB links mTORC1 signaling to transcriptional control of lysosome homeostasis. Sci Signal.

[35] Settembre C (2012). A lysosome-to-nucleus signalling mechanism senses and regulates the lysosome via mTOR and TFEB. EMBO J.

[36] Li Y (2016). Protein kinase C controls lysosome biogenesis independently of mTORC1. Nat Cell Biol.

[37] Miller AJ, Levy C, Davis IJ, Razin E, Fisher DE (2005). Sumoylation of MITF and its related family members TFE3 and TFEB. J Biol Chem.

[38] Palmieri, M., Pal, R. & Sardiello, M. AKT modulates the autophagy-lysosome pathway via TFEB. *Cell Cycle*, 1–2, 10.1080/15384101.2017.1337968 (2017). 10.1080/15384101.2017.1337968 PMC5531618 28636416

[39] Nord AS (2006). The International Gene Trap Consortium Website: a portal to all publicly available gene trap cell lines in mouse. Nucleic Acids Res.

[40] Hansen J (2003). A large-scale, gene-driven mutagenesis approach for the functional analysis of the mouse genome. Proc Natl Acad Sci USA.

[41] Austin CP (2004). The knockout mouse project. Nat Genet.

[42] Cobellis G (2005). Tagging genes with cassette-exchange sites. Nucleic Acids Res.

[43] Venken KJ (2011). MiMIC: a highly versatile transposon insertion resource for engineering Drosophila melanogaster genes. Nat Methods.

[44] Schnutgen F (2005). Genomewide production of multipurpose alleles for the functional analysis of the mouse genome. Proc Natl Acad Sci USA.

[45] Siepel A (2005). Evolutionarily conserved elements in vertebrate, insect, worm, and yeast genomes. Genome Res.

[46] Cooper GM (2005). Distribution and intensity of constraint in mammalian genomic sequence. Genome Res.

